# Pyrrolizidine Alkaloid Extraction and Analysis: Recent Updates

**DOI:** 10.3390/foods11233873

**Published:** 2022-11-30

**Authors:** Sarah F. Al-Subaie, Abdullah M. Alowaifeer, Maged E. Mohamed

**Affiliations:** 1 Department of Pharmaceutical Sciences, College of Clinical Pharmacy, King Faisal University, Al-Ahsa 31982, Saudi Arabia; 2 Reference Laboratory for Food Chemistry, Saudi Food and Drug Authority (SFDA), Riyadh 11561, Saudi Arabia

**Keywords:** pyrrolizidine alkaloids, GC/MS, LC/MS, necic acid, necine, food security

## Abstract

Pyrrolizidine alkaloids are natural secondary metabolites that are mainly produced in plants, bacteria, and fungi as a part of an organism’s defense machinery. These compounds constitute the largest class of alkaloids and are produced in nearly 3% of flowering plants, most of which belong to the Asteraceae and Boraginaceae families. Chemically, pyrrolizidine alkaloids are esters of the amino alcohol necine (which consists of two fused five-membered rings including a nitrogen atom) and one or more units of necic acids. Pyrrolizidine alkaloids are toxic to humans and mammals; thus, the ability to detect these alkaloids in food and nutrients is a matter of food security. The latest advances in the extraction and analysis of this class of alkaloids are summarized in this review, with special emphasis on chromatographic-based analysis and determinations in food.

## 1. Introduction

Plants and their phytoeffective metabolites are used for medicinal purposes but are also an enormous source of toxic products. Alkaloids contribute considerably to the medicinal and pharmacological activity of natural products while they are also recognized for high potency, a narrow therapeutic index, and, therefore, their toxicity. Alkaloids are produced with high diversity in prokaryotes and eukaryotes and are biosynthesized by many species of bacteria, fungi, marine organisms, insects, plants, and animals [1,2,3].

Pyrrolizidine alkaloids (PAs) and their *N*-oxides are produced by many flowering plants for protection. Approximately 660 PAs have been characterized in more than 6000 plants, occurring more frequently in the Asteraceae, Boraginaceae, Fabaceae, and Orchidaceae families and to a lesser extent in the Poaceae, Lamiaceae, and Convolvulaceae families [4,5,6,7]. Additional important plant families that contain PAs are Compositae and Leguminosae. PAs and their derivatives are found in many genera, such as *Alkanna*, *Cynoglossum*, *Heliotropium*, *Lithospermum*, *Symphytum*, *Anchusa*, and *Borago* from the Boraginaceae family and *Brachyglottis*, *Senecio*, *Tussilago*, *Cineraria*, *Petasites*, and *Eupatorium* from the Asteraceae family [6]. Other import genera containing PAs include *Amsinckia*, *Crotalaria*, *Echium*, and *Trichodesma* [8]. Although PAs are a source of the pharmacological activity in many medicinal plants and are therefore used in folk medicine [9], the toxicity of this class of alkaloids to humans and many animals usually compromises the medicinal benefits.

In this review, different separation methods and chemical analysis of PAs are first presented, followed by a summary of the widest possible range of mass spectrometer specifications used for the analysis of this class of alkaloids.

## 2. PA Chemistry

**Figure 1:**
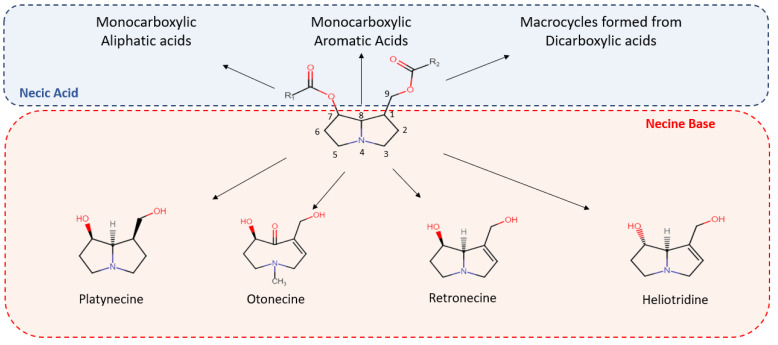
PAs are esters of necine and necic acids. Necine is a pyrrolizidine-based amino alcohol (the structure is shown in the red box) that exists in 4 different forms: platynecine, otonecine, retronecine, and heliotridine. Necic acid (the structure is shown in the upper blue box) exists as three different types: monocarboxylic (aliphatic and aromatic) and dicarboxylic acids separated or forming a macrocycle. One or both hydroxyl groups of necine can be esterified by necic acids, and there are also PAs that lack C7 oxygenation.

PAs are esters of necine alcohol and necic acids [9] and are described in Figure 1. Necine is a heterocyclic amino alcohol based on a pyrrolizidine nucleus containing two fused five-membered rings, including a nitrogen atom. Necine normally contains two hydroxyl groups, of which one is directly attached to the heterocycle and the other is attached to C1 via a hydroxymethyl group (Figure 1).

PAs are usually found in four different forms according to the N-oxidation and unsaturation levels of the pyrrolizidine ring; three of these forms are tertiary amine structures (saturated and unsaturated and otonecine) and the fourth is an *N*-oxide. PAs can be divided into different classes depending on the necine base, e.g., retronecine, heliotridine, otonceine, platynecine (Figure 1). Necic acids are a group of hydroxylated aliphatic acids containing either one or two carboxylic acid groups (Figure 1). Schramm, et al. [9] further classified PAs according to their overall structure into the following types: senecionine, triangularine, lycopsamine, monocrotaline, phalaenopsine/ipanguline, combined triangularine and lycopsamine, simple PAs, and PAs with unusual linkage patterns (more information can be found in [9]).

## 3. Toxicology of PAs

PAs are not intrinsically toxic; however, the 1,2-unsaturated PAs are metabolized in the liver into active pyrrolic metabolites, to which all the hepatotoxicity, including liver cirrhosis and liver failure, is attributed. As reported by Xia, et al. [10], the PA can lead to the formation of five different DNA reactive secondary pyrrolic metabolites. Moreover, it may cause pulmonary hypertension, cardiac hypertrophy, kidney degeneration, carcinogenicity, and genotoxicity, all of which could be fatal. [11,12,13]. The quantity and severity of the toxic metabolites produced by PAs results in different corresponding toxicity and potency levels (Table 1).

The ingestion of PAs is usually accompanied by toxicity symptoms ranging from nausea, vomiting, jaundice, and fever to hepatic occlusion [15]. According to the time and concentration of the exposure to PAs, alkaloid toxicity can be classified into chronic (long-term exposure with low concentrations of PAs) and acute (short-term exposure with high concentrations of PAs) toxicity, both of which can lead to serious illness, symptoms, and diseases in animals and humans.

## 4. Food and Pharmaceutical Products Safety Recommendation Regarding PAs

PAs and their *N*-oxide derivatives are found in many food products and supplements, particularly tea, herbal products, and honey. The European Food Safety Authority (EFSA) has identified a group of 17 PAs and their *N*-oxide derivatives that commonly contaminate food, including intermedine/lycopsamine, intermedine-*N*-oxide/lycopsamine-*N*-oxide, senecionine/senecivernine, senecionine-*N*-oxide/senecivernine-*N*-oxide, seneciphylline, seneciphylline-*N*-oxide, retrorsine, retrorsine-*N*-oxide, echimidine, echimidine-*N*-oxide, lasiocarpine, lasiocarpine-*N*-oxide, and senkirkine. To better understand the occurrence of PAs in food, PAs other than those mentioned in the 17-PAs list should also be monitored due to chromatographic coelution and structural isomerization problems [16]. As of July 2022, in Europe, the maximum PAs in different tea and herbal products came into effect, as shown in Table 2 [17].

EFSA recommends monitoring the concentration of these toxic alkaloids frequently to maintain the lowest possible occurrence in food chains [18]. Some countries, such as Germany, have introduced a limit of 1 μg/day of PAs for pharmaceutical products/medicines used for less than 6 weeks, and of 0.1 μg/day of PAs for consumption exceeding a 6-week period. Previously, the Federal Institute of Risk Assessment in Germany (BfR) recommended a daily intake of not more than 0.007 µg/kg body weight/day [15]. Furthermore, In 2017, and as a reference point for chronic risk assessment, the EFSA panel on contaminants chose a Benchmark Dose Lower Confidence limit for a 10% excess cancer risk (BMDL10) of 237 µg/kg BW per day for an increase in liver hemangiosarcoma incidence in female rats exposed to riddelliine [16].

## 5. Analysis of PAs

PA analysis can be divided into three phases: extraction, separation, and identification, the efficiency of which depends on many factors. Table 3 presents the most used gas and high-performance liquid chromatographic methods, including sample preparation, over the last 15 years.

### 5.1. PA Extraction

PA extraction from different samples depends on the form and type of the alkaloid of interest, as well as the complexity of the matrix used to implement the extraction process. The extraction process may involve three stages: sample preparation, PA extraction, and clean up. The preparation process can include simple cutting of a herbal product or homogenization/pulverization of frozen or dried material to increase the surface area for the extraction [83]. As shown in Table 3, the solid–liquid extraction is still the technique most widely used for sample preparation, although other extraction and purification techniques such as solid-phase extraction (SPE) or the QuEChERS procedure are being applied since they allow for cleaner extracts [84]. Extraction from differently prepared samples involves treatment with a specific solvent under suitable conditions to extract the maximum quality and quantity of the target alkaloids. All forms of PAs, including the *N*-oxides, have slight solubility in nonpolar solvents, i.e., hexane, and are therefore more efficiently extracted with polar solvents, such as methanol or with aqueous dilute acid; therefore, both methanol and dilute aqueous solutions of organic or mineral acids are good extraction solvents for PAs and their *N*-oxide derivatives [83]. Considering solubility effects, several techniques have been used to extract PAs from different matrices. Some examples of these extraction techniques are maceration [85], refluxing [86], percolation [87], sonication [88], Soxhlet-based extraction [89], supercritical fluid extraction [90], pressurized liquid extraction [91], microwave-assisted extraction [79], and solid phase extraction [92]. For example, These et al. [85] used 25% methanol in 2% formic acid for maceration in a single extraction process, followed by filtration or centrifugation [85]. El-Shazly et al. [93] homogenized herbal components in 0.5 N hydrochloric acid, followed by soaking for 1 h [93]. Mroczek et al. [87] extracted PAs by refluxing with 1% tartaric acid in methanol [87]. The extraction conditions can affect the quality and quantity of the PA yield, e.g., the temperature of the extraction can influence the extraction process; therefore, the prolonged use of Soxhlet extraction under a high reflux temperature has been found to result in a marked decrease in the PA yield [94].

A food matrix could be described as a complex assembly of nutrients and non-nutrients interacting physically and chemically. A food matrix could influences the release, mass transfer, and stability of many food compounds [95]; e.g., in terms of food analysis, there is variation between honey and tea or other herbal product, so a matrix should be considered when attempting to achieve effective extraction results. 

Solid phase extraction (SPE) techniques are another option for extracting and cleaning up PAs. The studies in Table 3 showed the utilization of SPE materials, e.g., Ergosil, C18-material, and strong cation exchange (SCX) for herbal products, including tea and spices, and illustrated that using SPE is necessary for many reasons, e.g., switching sample matrices to a form more compatible with chromatographic analyses, concentrating analytes for increased sensitivity, removing interferences to simplify chromatography and improve quantitation, and protecting the analytical column from contaminants. It is noted in most studies, as in Table 3, that there is a need to elute PAs and PA-*N*-oxides in SCX-based SPE with a basic solution, e.g., dilute NH_4_OH. 

### 5.2. PA Separation

PA separation is the main step after extraction. Many separation procedures can be used to analyze PAs, among which chromatographic techniques are currently the most utilized due to their ease of use and stability and reproducibility of results. Generally, the chromatographic separation and MS analysis of PAs and their *N*-oxides is a complex and complicated process owing to large numbers of structural and stereoisomers. This complexity and variation in the chemical structure enforced the utilization of many separations and isolation techniques in an attempt to solve the compound complexity matrix and reduce the problem of compound coelution. Examples of the separation techniques used are high-speed counter-current chromatography and capillary electrophoresis methods. Furthermore, detection techniques such as colorimetric, nuclear magnetic resonance-based, immunological-based, and UV-spectrometry-based or mass spectrometry-based techniques are now widely used to detect PAs, allowing the process of separation and detection and, therefore, sample preparation to be simpler and easier to apply [96]. The most efficient chromatographic techniques that were used to separate PAs were the liquid–gas, liquid–liquid, or liquid–solid techniques.

#### 5.2.1. PA Separation by Gas Chromatography

Table 3 shows examples of the most used gas chromatography methods for the analysis of PAs. PA *N*-oxides are not volatile and therefore cannot be detected by gas chromatography. Consequently (as shown in Table 3), in the reduction in PAs to their cores, retronecine and heliotridine, LiAlH_4_ is usually used as a reducing reagent. After reduction, the compounds are subjected to derivatization using N-methyl-N-(trimethylsilyl) trifluoroacetamide (MSTFA), heptafluorobutyric acid (HFBA), or other similar reagents. The inability to directly analyze PA *N*-oxides and the extensive preparation steps, including derivatization, causes the use of gas chromatography techniques to be impracticable for the analysis of PAs. Furthermore, reducing all PAs to their bases does not enable relative amounts of the original individual PAs and the *N*-oxides to be assessed.

#### 5.2.2. High-Performance Liquid Chromatography Separation of PAs

The use of high-performance liquid chromatography (HPLC), ultra-high-performance liquid chromatography (UHPLC), and liquid chromatography (LC) has been attracting an increasing interest for the separation of PAs, especially as LC–MS instruments become increasingly available (Table 3). LC–MS/MS methods have low detection limits (1 μg/kg or lower) and can be used to detect PAs and PA *N*-oxides simultaneously in a single run, as well as offering other advantages. Compared with GC, LC–MS offers the high-efficiency separation and detection of Pas without the need for derivatization, which means easier sample preparation. Even so, one of the main challenges in determining Pas or PA *N*-oxides by LC, HPLC, or UHPLC is the co-occurrence of isomers, which causes coelution, making it difficult to separate these compounds chromatographically and to identify them by mass spectrometry (since they have the same molecular weight and often very similar fragmentation patterns). Moreover, the disadvantage of these analysis techniques is the use of a targeted (non-broad-spectrum) setup, which could result in missing some PAs; furthermore, quantification necessitates the use of certified reference standards that are rare and very expensive [15,97]. Since targeted analysis focuses on specific compounds, it will not identify other compounds during analysis, so it is not effective for discovering new compounds or analyzing unknown samples [98]. In this case, nontarget analysis can reveal more broad information about new compounds [99]. An analysis of Table 3 indicated that the LC–MS methods can be used for both simple and complex matrices by slightly modifying the sample preparation methods to include a cleaning step. 

There are some PA isomers recommended to be monitored by the European Commission Regulation 2020/2040, e.g., indicine, echinatine, rinderine (possible coelution with lycopsamine/intermedine), indicine-*N*-oxide, echinatine-*N*-oxide, rinderine-*N*-oxide (possible coelution with lycopsamine-*N*-oxide/intermedine-*N*-oxide), integerrimine (possible coelution with senecivernine/senecionine), integerrimine-*N*-oxide (possible coelution with senecivernine-*N*-oxide/senecionine-*N*-oxide), heliosupine (possible coelution with echimidine), heliosupine-*N*-oxide (possible coelution with echimidine-*N*-oxide), spartioidine (possible coelution with seneciphylline), spartioidine-*N*-oxide (possible coelution with seneciphylline-*N*-oxide), usaramine (possible coelution with retrorsine), and usaramine *N*-oxide (possible coelution with retrorsine *N*-oxide) [47]. Chromatographic resolution is fundamental for the differentiation of isomeric PAs such as intermedine, indicine, lycopsamine, rinderine, and echinatine (*m*/*z* 300) and their *N*-oxides (*m*/*z* 316) as well as integerrimine, senecionine, and senecivernine (*m*/*z* 336) and their *N*-oxides (*m*/*z* 352), [100]. Klein, et al. [100] applied different acidic and alkaline mobile phases and succeeded to differentiate between some of the PA isomers, especially when alkaline conditions were applied. In the same study, the dimension of the C_18_ column and its particle size affected the resolution of the PA peaks produced. When a shorter column was used, this allowed for the reduction in sample size and produced a better separation and higher peak resolution. The problem of PA isomer separation will continue to be the most important problem in the analysis of PAs with only partial solutions, which allow for the separation and differentiation of particular groups of these alkaloids. 

### 5.3. PA Identification

Colorimetric, nuclear magnetic resonance-based (NMR), immunological, UV-spectrometry-based, and capillary electrophoresis methods have been used to analyze PAs as detection techniques, and NMR is used for structure identification [83] as well. The identification of PAs separated by LC procedures using MS-generated data remains challenging due to the high diversity and relative complexity of PA structures. Many characteristic mass fragments for the different types of PAs have been determined (Table 4) [85]. For example, Joosten, et al. [101] described the pyrrolizidines in *Jacobaea vulgaris* where 25 PAs were identified based on typical mass spectral transitions and retention time [101]. Lu et al. [102] performed a study on pyrrolizidines in the *Senecio* species and identified two mass ions at *m*/*z* 120 and 138 indicating the presence of retronecine-type PAs, as well as fragments at *m*/*z* 122, 150, and *m*/*z* 168 distinguishing otonecine-type PAs. Lu et al. [102] also identified fragments 122, 140 *m*/*z* as characteristic for the platynecine type of PAs. Moreover, PA *N*-oxides were found to produce a neutral fragment at *m*/*z* 44 [102]. Zhou et al. [103] developed a coupled precursor ion scan (PIS) and multiple reaction monitoring (MRM) approach to improve PA identification. Ruan et al. [104] studied the fragmentation pattern of some PA *N*-oxides and their related PAs. Retronecine-type PA *N*-oxides were found to produce two characteristic fragment clusters at *m*/*z* 118–120 and 136–138, which were not detected in the parent retronecine-type PAs. Likewise, fragmentation of the platynecine-type PA *N*-oxides was found to produce two characteristic ion clusters at *m*/*z* 120–122 and 138–140.

## 6. Conclusions

Pyrrolizidine alkaloids are compounds with different toxicity symptoms that should be detected in food and feed materials. PAs can be extracted similarly to other members in the class of alkaloids by acid–base, liquid–liquid, or liquid–solid extraction. Different techniques can be used to separate PAs and their *N*-oxides, of which the most common are LC–MS or GC–MS. GC–MS cannot be used to identify PA *N*-oxides directly and requires extensive sample preparation; consequently, GC–MS is generally considered to be impracticable for PA separation. On the other hand, LC–MS and LC–MS/MS are currently the most applied techniques for the separation and identification of PAs and their *N*-oxides because of numerous advantages, including effective separation, the potential for a wide range of compounds to be identified, and simple sample preparation. Nowadays, there are methods for detecting and identifying PAs from MS/MS traces, but these methods still need to be improved in the future in order to reduce the time and to distinguish between PA isomers more accurately. On the other hand, nontargeted PA detection needs more development to increase the specificity and sensitivity of the process to more accurately identify these alkaloids. Further clinical studies are recommended to assess the pharmacodynamic and pharmacokinetic effects of Pas on humans and animals in more detail. Finally, studies on Pas require a high safety level and detailed analyses.

## Figures and Tables

**Table 1:** In silico predicted lethal dose 50 (LD_50_) values of some PAs [14].

PA	LD_50_ (g/kg)
Monocrotaline *	0.731
Echimidine	0.616
Senkirkine	0.275
Trichodesmine	0.324
Acetyllycopsamine	0.356
Seneciphylline	0.264
Retrorsine *	0.320
Senecionine	0.127
Heliosupine	0.708
Riddelliine	0.616
Clivorine	0.386
Usaramine	0.264
Jacobine	0.461
Echiumine	0.122
Lycopsamine	0.239
Heliotrine	0.056
Heliocoromandaline	0.246
Otosenine	0.106

* In vitro test compound.

**Table 2:** European Commission Regulation for the maximum sum level of the 21 PAs, together with the other 14 coeluting PAs for certain foodstuffs [17].

Foodstuffs	Max Sum Level of PAs (µg/kg)
Herbal infusions (dried product)	200
Herbal infusions of rooibos, anise (*Pimpinella anisum*), lemon balm, chamomile, thyme, peppermint, lemon verbena (dried product), and mixtures exclusively composed of these dried herbs	400
Tea (*Camellia sinensis*) and flavored tea (*Camellia sinensis*) (dried product)	150
Tea (*Camellia sinensis*), flavored tea (*Camellia sinensis*), and herbal infusions for infants and young children (dried product)	75
Tea (*Camellia sinensis*), flavored tea (*Camellia sinensis*), and herbal infusions for infants and young children (liquid)	1.0
Food supplements containing herbal ingredients including extracts	400
Pollen-based food supplements, pollen, and pollen products	500
Borage leaves (fresh, frozen) placed on the market for the final consumer	750
Cumin seeds (seed spice)	400
Borage, lovage, marjoram, and oregano (dried) and mixtures exclusively composed of these dried herbs	1000

**Table 3:** Separation methods of PAs in last 15 years (2007–2022).

Sample		Analysis				
Sample Type	Sample Preparation	Instrument	Analytes	Recovery (%)	LOD/LOQ	Ref.
Honey and herbal beverage	Prepare using QuEChERs Solvent for extraction: 1 mL water followed by 5 mL ACN Partition salts: 1 g NaCL Clean-up process: SPE using 50 mg PSA	UPLC-IM-QTOF-MS/MS Mode: +Ve ESI Column: C18 (2.1 mm × 100 mm; 1.7 μm; Waters) at 50 °C M.P: A: 0.1% FA in H_2_O, B: 0.1%FA in ACN	7 PAs	61–120	LOQ: 1–20 µg/kg	[19]
Teas and herbs	Prepare using QuEChERs Solvent for extraction: 30 mL ACN: water (75:25, *v*/*v*) with 0.5% FA Partition salts: 6 g MgSO_4_ and 1.5 g CH_3_COONa Clean-up process: SPE using 400 mg PSA, 400 mg C18, 400 mg GCB, and 1200 mg MgSO_4_	HPLC-Q-Orbitrap-MS/MS Mode: +Ve ESI, HRMS Column: C18 at 40 °C	28 Pas/PA *N*-Oxides	87–111	LOQ: 5 µg/kg	[20]
Aromatic herbs	Prepare using QuEChERs Solvent for extraction: 1 mL H_2_O followed by 1 mL ACN Partition salts: 0.4 g MgSO_4_, 0.1 g TSCDH, 0.05 g DSHCSH, and 0.1 g NaCl Clean-up process: 25 mg LP-MS-NH_2_ and 150 mg MgSO_4_	UHPLC-IT-MS/MS Mode: +Ve ESI, MRM Column: C18 at 25 °C	21 PAs/PA *N*-Oxides	73–105	LOQ: 1.2–9.9 µg/kg	[21]
Pollen	Prepare using QuEChERs Solvent for extraction: 10 mL H_2_SO_4_ (0.1 M) Partition salts: 4 g MgSO_4_, 1 g TSCDH, 0.5 g DSHCSH, and 1 g NaCl Clean-up process: 150 mg PSA and 900 mg MgSO_4_	UHPLC-TQ-MS/MS Mode: +Ve ESI, MRM Column: RP-MS at 40 °C	20 PAs	73–106	LOQ: 4.0–9.0 µg/kg	[22]
Teas and Weeds	Solvent for extraction: 0.1 M of H_2_SO_4_ Clean-up process: PCX-SPE Elution solvent: MeOH + 0.5% NH_4_OH	UHPLC-MS/MS Mode: +Ve ESI, MRM Column: UPLC HSS T3 (100 × 2.1 mm id, 1.8 μm) at 40 °C M.P: A: 0.1% FA in MeOH, B: 0.1%FA in H_2_O	14 PAs/PA *N*-Oxides	68–110	LOD: 0.001–0.4 μg/kg LOQ: 1–5 μg/kg	[23]
Honey	Solvent for extraction: 6.5 mmol/L NH_4_OH Clean-up process: filter through 0.22 μm PVDF	UHPLC-QTOF-MS/MS Mode: +Ve ESI, HRMS Column: BEH C18 (100 × 2.1 mm, 1.7 µm) at 50 °C M.P: A: 6.5 mmol/L NH_4_OH in H_2_O, B: 6.5 mmol/L NH_4_OH in ACN	26 PAs/PA *N*-Oxides	75–120	LOD: 1–7 µg/kg LOQ: 10–20 µg/kg	[24]
Herbal Medicines	Solvent for extraction: 50% MeOH + 0.05 M H_2_SO_4_ Clean-up process: MCX—SPE	LC-MS/MS Mode: +Ve ESI, MRM Column: C18 (2.1 mm, 150 mm, 2 µm), at 40 °C M.P: A: 0.1% FA in 5 mM NH_4_HCO_2_, B: 0.1% FA in 5 mM NH_4_HCO_2_ in 100% MeOH	28 PAs	67–151	LOD: 0.03–2.1 µg/kg LOQ: 0.1–6.5 µg/kg	[25]
Black tea and Herbal tea	Solvent for extraction: 50% MeOH solution with 0.05 M H_2_SO_4_ Clean-up process: MCX—SPE Elution solvent: 4 mL of 2.5% ammonia in MeOH	UPLC-MS/MS Mode: +Ve ESI, MRM Column: C18 (100 mm, 2.1 mm, 3.5 µm) at 40 °C M.P: A: 0.1% FA in 5 mM NH_4_HCO_2_, B: 0.1% FA in 5 mM NH_4_HCO_2_ in 95% MeOH	21 PAs	86–101	LOD: 0.1–3 µg/kg LOQ: 0.3–9 µg/kg	[26]
Milk	Solvent for extraction: LLE with 0.5% FA; then LLE with DCM	DART-IT-MS Mode: +Ve Column: C18 (1.7 μm, 2.1 mm × 100 mm) M.P: A: 0.1% FA in H_2_O, B: ACN	6 PAs	89–112	LOD: 0.5–8 µg/kg LOQ: 1.8–2.8 µg/kg	[27]
Dried Plant, Pollen, and Honey	Plant and pollen: Solvent for extraction: 70% MeOH in H_2_O acidified with 2% FA	LC-Q-TRAP-MS/MS Mode: ESI, MRM Column: C18 (3.0 mm × 100 mm × 3.5 μm at 30 °C M.P: A: H_2_O + 0.1% FA, B: ACN + 0.1% FA	8 PAs/PA *N*-Oxides	-	-	[28]
Honey	Solvent for extraction: LLE + 0.05 M H_2_SO_4_ Clean-up process: SCX-SPE	HPLC-DAD (wavelength: 223 nm) Column: C18 (250 × 4.6 mm; 5 μm) M.P: A: H_2_O + H_3_PO_4_, B: ACN: H_2_O (90:10, *v*/*v*)	2 PAs	-	-	[29]
Honey	Solvent for extraction: LLE + 0.05 M H_2_SO_4_: MeOH (85:15, *v*/*v*) Clean-up process: MCX-SPE	HPLC-TQ-MS/MS Mode: +Ve ESI, MRM Column: C18 (2.1 × 100 mm, 1.7 mm) M.P: H_2_O: ACN, 85:15	17 PAs	-	-	[30]
Maize	Solvent for extraction: SLE + 0.05 M H_2_SO_4_ Clean-up process: SCX-SPE	HPLC-QTRAP-MS/MS Mode: +Ve ESI, MRM Column: C12 (150 mm × 2.1 mm, 4 mm) at 40 °C M.P: A: 0.3% FA in H_2_O, B: 0.3% FA in ACN	Sum of 1, 2- unsaturated retronecine/ heliotridine- PAs	-	-	[31]
Plant and Seeds	Solvent for extraction: 2% FA in MeOH using MSDP Clean-up process: filtered through (0.22 μm–PTFE) filter	UHPLC-MS/MS Mode: +Ve ESI, MRM Column: C18 (100 mm × 2.1 mm id, 1.9 μm) At 30 °C M.P: A: FA/H_2_O, B: FA/ACN, different FA concentrations (0.05, 0.2, and 0.35% *v*/*v*) were used	45 PAs/PA *N*-oxides		LOD: 0.05 ng/mL LOQ: -	[32]
Oregano	Prepare using QuEChERs Solvent for extraction: 1 mL H_2_O followed by 1 mL ACN Partition Salts: 0.4 g MgSO_4_, 0.1 g TSCDH, 0.05 g DSHCSH, and 0.1 g NaCl Clean-up process: 25 mg PSA and 150 mg MgSO_4_	UHPLC-IT MS/MS Mode: +Ve ESI, MRM Column: C18 (100 mm × 2.1 mm, 1.6) at 25 °C M.P: A: 0.2% FA + 5 mM CH_3_COONH_4_ in H_2_O, B: 10 mM CH_3_COONH_4_ in MeOH	21 PAs/PA *N*-oxides	77–96	LOD: 0.1–7.5 µg/kg LOQ: 0.5–25.0 µg/kg	[33]
Spices and Herbs	Solvent for extraction: SLE + 0.05 M H_2_SO_4_ Clean-up process: SCX-SPE	HPLC-TQ-MS/MS Mode: +Ve ESI, MRM Column: C18 (2.5 μm, 100 Å, 100 × 30 mm) M.P: A: 0.1% FA in H_2_O, B: 0.1% FA in ACN	44 PAs/PA *N*-oxides	50–119	LOD: Less than 0.1–2.6 µg/kg LOQ: -	[5]
Herbs	Prepare using QuEChERs Solvent for extraction: add 10 mL H_2_O, then add 10 mL ACN with 1% FA Partition salts: 4 g MgSO_4_, 1 g TSCDH, 0.5 g DSHCSH, and 1 g NaCl Clean-up process: 200 mg graphene	HPLC-QTRAP MS/MS Mode: +Ve ESI, MRM Column: C18 (100 × 2.1 mm, 1.9 µm) at 40 °C M.P: A: H_2_O, B: ACN	30 PAs/PA *N*-oxides	61–128	LOD: - LOQ: 1 µg/kg	[34]
Herbs	Solvent for extraction: MeOH: H_2_O: FA, 60:39.6:0.4, *v*/*v*/*v* Clean-up process: SPE	UHPLC-QTRAP-MS/MS Mode: +Ve ESI, MRM Column: HILIC (150 × 2.1 mm; 1.6 μm) at 40 °C M.P: A: H_2_O, B: 5 mM of NH_4_HCO_2_ + 0.1% FA in ACN:H_2_O (95:5, *v*/*v*)	33 PAs/PA *N*-oxides	78–117	LOD: - LOQ: 0.5–10 µg/kg	[35]
Honey	Prepare using QuEChERs Solvent for extraction: 10 mL H_2_O then 10 mL ACN Partition salts: 4 g MgSO_4_ and 1 g NaCl Clean-up process: -	LC-QTRAP MS/MS Mode: +Ve ESI, MRM Column: C18 (150 × 4.6 mm, 5 μm) at 18 °C M.P: 0.1% FA in H_2_O, B: ACN	5 PAs/PA *N*-oxides	86–111	LOD: - LOQ: 8–18 µg/kg	[36]
Honey	Solvent for extraction: DLLME + CHCl_3_ and iPrOH	UHPLC-QTRAP-MS/MS Mode: +Ve ESI, MRM Column: C18 (100 × 2.1 mm i.d., 1.6 μm) at 30 °C M.P: A: 0.1% HCOOH in H_2_O, B: 0.1% HCOOH in ACN	9 PAs/PA *N*-oxides	63–103	LOD: - LOQ: 0.03–0.06 µg/kg	[37]
Herbal teas	Solvent of extraction: boiling water for infusion	UHPLC-TQ-MS/MS Mode: +Ve MRM Column: C18 (150 × 2.1 mm, 1.7 μm) at 50 °C M.P: 10 mM of (NH_4_)_2_CO_3_ in H_2_O, B: ACN	70 PAs/PA *N*-oxides	73–107	LOD: 0.01–0.02 µg/L LOQ: 0.05 µg/L	[38]
Herbal juices	Solvent of extraction: LLE + 0.05 M of H_2_SO_4_ Clean-up process: SCX-SPE	HPLC-QTRAP-MS/MS Mode: +Ve ESI, MRM Column: C18 at 25 °C	30 PAs/PA *N*-oxides	-	-	[39]
Honey	Solvent of extraction: LLE + 0.05 M of H_2_SO_4_ Clean-up process: MCX-SPE	HPLC-Q-TOF-MS/MS Mode: +Ve ESI, HRMS Column: C18 (2.7 μm, 100 × 2 mm) at 40 °C M.P: A: 0.1% FA + 2.5 mM NH_4_OH in H_2_O, B: ACN	12 PAs/PA *N*-oxides	79–104	LOD: 0.2–0.6 µg/kg LOQ: 0.5–1.3 µg/kg	[40]
Herbs	Solvent of extraction: SLE + 2% FA	HPLC-TQ-MS/MS Mode: +Ve ESI, MRM Column: C18 (150 × 2.0 mm, 3µm) M.P: A: H_2_O, B: ACN	12 PAs/PA *N*-oxides	-	-	[41]
Teas and Herbs	Solvent of extraction: SLE + 0.05 M of H_2_SO_4_ Clean-up process: SCX-SPE	HPLC-TQ-MS/MS Mode: +Ve ESI, MRM Column: C18 (150 × 2.1 mm, 5 µm) at 30 °C M.P: A: H_2_O, MeOH/H_2_O (10/90, 5/95, *v*/*v*) or ACN/H_2_O (10/90, 5/95, *v*/*v*), B: MeOH/H_2_O or ACN/H_2_O (95/5 *v*/*v*) or (90/10 *v*/*v*), C: MeOH/H_2_O (90/10, *v*/*v*) or ACN/H_2_O (90/10, *v*/*v*)	44 PAs/PA *N*-oxides	52–152	LOD: 0.1–7.0 µg/kg LOQ: 0.1–27.9 µg/kg	[42]
Milk, Dairy products, eggs, meat, meat products, Herbs and Food supplements	Animal-derived samples: Solvent of extraction: LLE or SLE + 0.2% FA solution + hexane Clean-up Process: MCX-SPE Herbal samples: Solvent of extraction: infusion with boiling water Clean-up process: MCX-SPE Supplements: Solvent of extraction: SLE + 0.05 M H_2_SO_4_ Clean-up process: MCX-SPE Oily food supplements: Solvent of extraction: SLE + 0.05 M of H_2_SO_4_ Clean-up process: SCX-SPE	UHPLC-TQ-MS/MS Mode: +Ve ESI, MRM Column: C18 (150 × 2.1 mm, 1.7 μm) at 50 °C M.P: A: H_2_O + 6.5 mM NH_4_OH, B: ACN + 1.2 mM NH_4_OH	38 PAs/PA *N*-oxides	30–122	LODs: Milk and yoghurt 0.03–0.05 µg/L egg, cheese, chicken, and pork meat: 0.05–0.15 µg/kg red meat: 0.1–0.25 µg/kg Teas and supplements: 0.2–3.8 µg/kg	[43]
Honey	Solvent of extraction: LLE + 0.15 M HCl Clean-up process: MCX-SPE	GC-Q-MS EI Carrier gas: helium Column: capillary column (30 m × 0.25 mm, 0.25)	4 PAs/PA *N*-oxides	73–94	LOD: - LOQ: 1 µg/kg	[44]
Herbs, Spices, Teas, and ice-tea drinks	Herbs: Solvent of extraction: SLE + 0.1% FA in MeOH Clean-up process: SPE Infusion extracts and ice-tea drinks: Solvent of extraction: infusion of teas with boiling water Clean-up process: MCX-SPE	UHPLC-TQ-MS/MS Mode: +Ve ESI, MRM Column: C18 (100 × 2.1 mm,1.7 μm) at 45 °C M.P: A: 0.1% NH_3_ in H_2_O, B: ACN	31 PAs/PA *N*-oxides	86–125	No LODs for all LOQs: 0.1–1 ng/g Infusion extracts: 0.01 ng/mL	[45]
Peppermint tea and Honey	Solvent of extraction: SLE or LLE + 0.05 M of H_2_SO_4_ Clean-up process: SCX-SPE	HPLC-TQ-MS/MS Mode: +Ve ESI, MRM Column: C8 (150 × 2.0 mm, 4 μm) at 30 °C M.P: A: 0.1% FA + 5 mmol/L NH_4_HCO_2_ in H_2_O, B: 0.1% FA + 5 mmol/L NH_4_HCO_2_ in MeOH	25 PAs/PA *N*-oxides	49–121	LOD: 0.01–1.60 µg/kg LOQ: 0.03–5.40 µg/kg	[46]
Honey	Extract using QuEChERS Solvent of extraction: LLE with 10 mL H_2_SO_4_ (0.1 M), add zinc dust, then supernatant with 10 mL ACN Partition salts: 4 g MgSO_4_, 1 g TSCDH, 0.5 g DSHCSH, and 1 g NaCl Clean-up process: 150 mg PSA and 900 mg MgSO_4_	HPLC-Q-Orbitrap-MS/MS Mode: +Ve ESI, HRMS Column: C8 (150 × 3 mm, 2.7 μm) at 35 °C M.P: A: 0.1% FA in H_2_O, B: MeOH: ACN 1:1 *v*/*v*	9 PAs/PA *N*-oxides	92–115	LOD: 0.04–0.2 µg/kg LOQ: 0.1–0.7 µg/kg	[47]
Plants	Solvent of extraction: LLE using CHCl_3_/MeOH (85:15), then add 5 mL of NH_4_OH (25% solution) Clean-up process: Add 2 M of HCl to extract then neutralize the aqueous layer with Na_2_CO_3_ and extract with CHCl_3_.	GC-MS Carrier gas: helium Column: capillary column (15 m; 0.25 mm i. d.; 0.25 μm)	5 PAs	-	-	[48]
Feed (Silage and hay)	Solvent of extraction: 1 M HCl, then pH adjusted to 10–11 with NH_3_ Clean-up process: SPE	GC-MS Carrier gas: helium Column: capillary column (30 m × 0.25 mm, 0.25)	2 (sum of retronecine derivative and heliotridine derivative)	72.7–94.4	LOD: - LOQ: 10 µg/kg	[49]
Honey	Clean-up process: SCX-SPE	LC-IT-MS/MS Mode: +Ve ESI, MRM Column: C18 (100 × 2.1 mm, 1.9 μm) M.P: A: 0.1% FA in H_2_O, B: 0.1% FA in MeOH	6 PAs/PA *N*-oxides	74–108	LOD: - LOQ: 0.25 µg/kg	[50]
Honey	Solvent of extraction: dilution by distilled water only Clean-up process: -	HPLC-QTRAP-MS/MS Mode: +Ve ESI, MRM Column: C18 (100 mm × 3.0 mm; 3.5-μm) M.P: A: 0.1% FA in H_2_O, B: 0.1% FA in ACN	8 PAs/PA *N*-oxides	93–110	LOD: 0.1–1 µg/kg LOQ: 0.2–1.5	[51]
Eggs and Meat	Solvent of extraction: SLE by 0.2% FA and hexane, then NH_3_ pH is adjusted to 9.0–10.0 Clean-up process: MCX-SPE	UHPLC-TQ-MS/MS Mode: +Ve ESI, MRM Column: C18 (150 × 2.1 mm, 1.7 μm) at 50 °C M.P: 6.5 mM NH_3_ in ACN/H_2_O	51 PAs/PA *N*-oxides	-	LOD: - LOQ: 0.1–1 µg/kg	[52]
Milks, Soybean, Seed oils, and Margarines	Milk and Soy: Solvent of extraction: SLE or LLE using CHCl_3_:MeOH (1:1, *v*/*v*), Clean-up process: SCX-SPE Seed oils and margarine: Solvent of extraction: SLE or LLE by MeOH Clean-up process: SCX-SPE	HPLC-TQ-MS/MS Mode: +Ve ESI, MRM Column: C18 (150 × 4.6 mm, i.d., 3 μm) at 30 °C M.P: A: 0.1% FA in H_2_O, B: ACN	9 PAs/PA *N*-oxides	82–105	LOD: 0.07–0.59 µg/kg LOQ: 0.20–1.43 ng/mL	[53]
Herbal supplements	Solvent of extraction: SLE using MeOH Clean-up process: -	UHPLC-Q-TOF-MS/MS Mode: +Ve ESI, all ion MS/MS mode Column: C18 (2.1 × 150 mm, 2.7 μm) at 40 °C M.P: A: 0.1% FA in H_2_O, B: 0.1% FA in ACN	25 PAs/PA *N*-oxides	-	LOD: 0.05–5 ng/mL LOQ: -	[54]
Teas, Wheat, and Leek	Prepare using QuEChERs Solvent of extraction: acidification with 10 mL water with 0.2% FA, followed by 10 mL ACN Partition salts: 4 g MgSO_4_ and 1 g NaCl Clean-up process: 100 mg C18 and 300 mg MgSO_4_	HPLC-Q-Orbitrap-MS/MS Mode: ESI +Ve and, ESI −Ve HRMS Column: C8 (150 mm × 2.1 mm i.d., 2.6 mm) at 25 °C M.P: for +Ve ESI, A: 0.1% FA + 5 mM NH_4_HCO_2_ in H_2_O, B: 0.1% FA + 5 mM NH_4_HCO_2_ in MeOH, for −Ve ESI, A: 5 mM of NH_4_CH_3_CO_2_ in H_2_O, B: 5 mM of NH_4_CH_3_CO_2_ in MeOH	11 PAs/PA *N*-oxides	71–93	LOD: - µg/kg LOQ: 1–100 µg/kg	[55]
Herbal teas	Dry samples: Solvent of extraction: SLE + 0.05 M of H_2_SO_4_, then using NH_3_ pH is adjust to 6.0–7.0 Clean-up process: MCX-SPE Infusion samples: Solvent of extraction: infusion by boiling water Clean-up using: MCX-SPE	HPLC-TQ-MS/MS Mode: +Ve ESI, MRM Column: C18 (150 × 2.1 mm; 1.9μm) at 20 °C M.P: A: 0.1% FA + 5 mM NH_4_HCO_2_ in H_2_O, B: 0.1% FA + 5 mM NH_4_HCO_2_ in MeOH	23 PAs/PA *N*-oxides	76–125	LOD: - LOQ: 10 µg/kg	[56]
Honey	Solvent of extraction: LLE + 0.05 M of H_2_SO_4_ Clean-up process: SCX-SPE	HPLC-TQ-MS/MS Mode: +Ve ESI MRM Column: PFP (150 × 2.1 mm, 2.6 μm) at 35 °C M.P: A: 95:5 *v*/*v* H_2_O/ACN + 0.05% FA, B: 100% ACN	14 PAs/PA *N*-oxides	82–112	LOD: 0.4–3.3 µg/kg LOQ: 1.4–10.9 µg/kg	[57]
Honey	Solvent of extraction: LLE + 0.05 M of H_2_SO_4_ Clean-up process: SCX-SPE	HPLC-TQ-MS/MS Mode: +Ve ESI, SIM Column: C18 (100 × 30 mm, 2,5 µm) at 25 °C M.P: A: 0.1% FA in H_2_O, B: 0.1% FA in ACN	5 PAs/PA *N*-oxides	40–106	LOD: 0.45–0.67 ng/mL LOQ: 1.21–1.79 ng/mL	[58]
Feed	Prepare using QuEChERs Solvent of extraction: 10 mL ACN followed by 10 mL 0.1% FA in H_2_O Partition salts: 4 g MgSO_4_ and 1 g NaCl Clean-up process: -	UHPLC-TQ-MS/MS Mode: +Ve ESI, MRM Column: C18 (50 mm × 2.1 mm,1.7 µm) at 40 °C M.P: A: 0.1% FA in H_2_O, B: 0.1% FA + 1 mM NH_4_HCO_2_ in MeOH	5 PAs	72–98	LOD: - LOQ: 5 µg/kg	[59]
Honey	Solvent of extraction: LLE + 0.05 M of H_2_SO_4_ Clean-up process: SCX-SPE	HPLC-TQ-MS/MS Mode: +Ve MRM Column: C18 (100 × 2.1 mm i.d., 1.9 μm) at 30 °C M.P: A: 0.05% FA in H_2_O, B: 100% ACN	14 PAs/PA *N*-oxides	70–125	LOD: 0.5–3.9 µg/kg LOQ: 2.3–12.9 µg/kg	[60]
Herbal teas	Solvent of extraction: SLE with aqueous AcOH: MeOH (1:2, *v*/*v*), then NH_3_ (till pH 5.0–6.0) Clean-up process: -	HPLC-QTRAP-MS/MS Mode: +Ve MRM Column: C18 (50 × 2.1 mm, 1.9 mm) at 25 °C M.P: A: 0.5% FA in H_2_O, B: 94.5% MeOH, 5% H_2_O and, 0.5% FA	28 PAs/PA *N*-oxides	80–95	LOD: - LOQ: 10–50 µg/kg	[61]
Herbal teas	Solvent of extraction: SLE + 0.05 M of H_2_SO_4_ Clean-up process: SCX-SPE	HPLC-TQ-MS/MS Mode: +Ve ESI, MRM Column: PFP (150 × 2.1 mm, 2.6 μm) at 35 °C M.P: A: 0.05% FA + 5% ACN in H_2_O, B: 100% ACN	14 PAs/PA *N*-oxides	93–127	LOD: 0.4–1.9 µg/kg LOQ: 1.3–6.3 µg/kg	[62]
Eggs	Solvent of extraction: SLE + 0.05 M of H_2_SO_4_ + ACN Clean-up process: SCX-SPE	HPLC-IT-MS/MS Mode: +Ve ESI Column: C8 (150 × 2.0 mm) M.P: A: 100% ACN, B: 0.1 FA ACN	2 PAs/PA *N*-oxides	-	LOD: - µg/kg LOQ: 2 ng/g	[63]
Honey	Prepare using QuEChERs Solvent of extraction: LLE + 10 mL H_2_SO_4_ (0.05 M), add zinc dust, supernatant with 10 mL ACN Partition salts: 4 g MgSO_4_, 1 g TSCDH, 0.5 g DSHCSH, and 1 g NaCl Clean-up process: 150 mg PSA, 45 mg C18, and 900 mg MgSO_4_	UHPLC-Q-MS Mode: +Ve ESI, SIM Column: C8 (15 cm × 3 mm, 2.7 mm) at 34 °C M.P: A: 0.5% FA in H_2_O, B: 100% ACN	9 PAs	67–122	LOD: - LOQ: 0.08–4.3 µg/kg	[64]
Honey	Solvent of extraction: LLE + 0.05 M of H_2_SO_4_ Clean-up process: SCX-SPE	HPLC-QTRAP-MS/MS Mode: +Ve MRM Column: C18 (50 × 2.1 mm, 1.9 μm) at 25 °C	18 PAs/PA *N*-oxides	-	LOD: - LOQ: 1–3 µg/kg	[65]
Honey and Herbal teas	Honey Samples Solvent of extraction: LLE + 0.05 M of H_2_SO_4_ Clean-up process: SCX-SPE Herbal Teas Solvent of extraction: SLE + 0.05 M of H_2_SO_4_, then using NH_3_ (till pH 6.0–7.0) Clean-up process: reversed phase-SPE	HPLC-TQ-MS/MS Mode: +Ve ESI, MRM Column: C18 150 × 2.1 mm, 1.9 µm) M.P: A: 0.1% FA + 5 mM NH_4_HCO_2_ in H_2_O, B: 95% MeOH + 5% H_2_O containing 0.1% FA + 5 mM NH_4_HCO_2_	17 PAs/PA *N*-oxides	45–122	LOD: 0.06–2.0 µg/kg LOQ: 0.18–6.4 µg/kg	[66]
Herbal supplement in form of tablets, capsules, soft gels, and liquids	Prepare using QuEChERs Solvent for extraction: Tablets and capsules: 10 mL deionized water with 2% FA, afterward 10 mL ACN. Soft gels: defatted with 4 mL hexane, add 10 mL deionized water with 2% FA, afterward 10 mL ACN. Liquids: 10 mL ACN + 2% FA Partition salts: 4 g MgSO_4_ and 1 g NaCl Clean-up process: 100 mg C18 silica and 300 mg MgSO_4_	UHPLC-Q-Orbitrap-MS/MS Mode: +Ve ESI, HRMS Column: HSS T3 (100 mm × 2.1 mm i.d., 1.8 µm) at 40 °C M.P: A: 0.1% FA + 5 mM NH_4_HCO_2_ in H_2_O, B: M.P: A: 0.1% FA + 5 mM NH_4_HCO_2_ in MeOH	11 PAs/PA *N*-oxides	70–120	LOD: - LOQ: 50–2500 µg/kg	[67]
Honey	Solvent of extraction: LLE + 0.5 M of H_2_SO_4_ Clean-up process: SCX-SPE	HPLC-TQ-MS/MS Mode: +Ve ESI, MRM Column: C18 (150 × 2.1 mm i.d) M.P: A: 0.1% FA in H_2_O, B: 0.1% FA in MeOH	17 PAs/PA *N*-oxides	More than 80%	LOD: - LOQ: 1–3 µg/kg	[68]
Honey	Solvent of extraction: LLE + 0.05 M of H_2_SO_4_ Clean-up process: SCX-SPE	HPLC-IT-MS/MS Mode: +Ve ESI Column: C18 (150 × 2.1 mm, 3 µm) at 30 °C M.P: A: 0.05% FA in H_2_O, B: 100% ACN	11 PAs/PA *N*-oxides	87	LOD: 0.01–0.03 µg/mL LOQ: 0.04–0.10 µg/kg	[69]
Honey and mead	Honey: Solvent of extraction: LLE with MeOH Clean-up process: SCX-SPE Mead: Solvent of extraction: 0.05 M of H_2_SO_4_ pH adjusted to 1.6–2.7 Clean-up process: SCX-SPE	HPLC-IT-MS/MS Mode: +Ve ESI Column: C18 150 mm × 2.1 mm i.d., 4 µm) M.P: 0.1% FA in H_2_O	7 PAs/PA *N*-oxides	-	LOD: 50 ng/kg LOQ: -	[70]
Herbs and Honey	Solvent of extraction: LLE or SLE 0.05 M of H_2_SO_4_ Clean-up process: SCX-SPE	HPLC-QTRAP-MS/MS Mode: ESI, MRM Column: C18 (150 mm × 2.1 mm, 3 μm) M.P: A: 0.1 M FA in H_2_O, B: 100% ACN	3 PAs/PA *N*-oxides	69–104	LOD: 0.1–1 µg/kg LOQ: 0.3–3 µg/kg	[71]
Honey	Solvent of extraction: 0.05 M sulfuric acid, then add zinc and filtration using glass wool Clean-up process: SCX-SPE	HRGC-Q-MS Mode: +Ve SIM Column: ZB-5MS (30 m × 0.25 mm; ft 0.25 μm)	2 PAs/PA *N*-oxides	-	LOD: 2 µg/kg LOQ: 6 µg/kg	[72]
Milk	Solvent of extraction: 0.1% FA in MeOH for precipitation, followed by evaporation to concentration Clean-up process: -	UHPLC-QHQ-MS/MS Mode: +Ve MRM Column: C18 (150 ×2.1 mm, 1.7 mm) at 50 °C M.P: 6.5 mM of NH_3_ in ACN/H_2_O mixture	21 PAs/PA *N*-oxides	44–67	LOD: - LOQ: 0.05–0.2 µg/L	[73]
Honey, Food supplements, and feed	Prepare using QuEChERs Solvent of extraction: 10 mL H_2_O followed by 10 mL ACN with 1% AcOH Partition salts: 4 g MgSO_4_ and 1 g CH_3_COONa Clean-up process: -	HPLC-Orbitrap-MS Mode: +Ve and −Ve ESI Column: C18 (100 × 3 mm ID, 3 mm) at 35 °C M.P: A: 2 mM of NH_4_HCO_2_ + 0.5 mM FA in H_2_O, B: 2 mM NH_4_HCO_2_ + 0.5 mM FA in MeO:H_2_O, 95:5	14 PAs/PA *N*-oxides	-	-	[74]
Honey, pollen, and honey- products	Mead and fennel honey: Solvent of extraction: LLE + 0.05 M of H_2_SO_4_ Clean-up process: SCX-SPE Rest of foodstuff: Solvent of extraction: LLE with pentane: DCM (2:1, *v*/*v*) Clean-up process: SCX-SPE	HRGC-Q-MS Mode: EI and SIM Column: capillary column	6 PAs/PA *N*-oxides	74–88	LOD: - LOQ: 10 µg/kg	[75]
Honey	Prepare using QuEChERs Solvent of extraction: dilution with 4 mL H_2_O, followed by 4 mL ACN Partition salts: 0.8 g MgSO_4_, 0.2 g TSCDH, 0.1 g DSHCSH, and 0.2 g NaCl Clean-up process: dSPE (500 mg MgSO_4_)	HPLC-TQ-MS/MS Mode: +VE ESI Column: C18 (150 × 2.1 mm, 5 mm) M.P: A: 0.1% CH_3_COOH in H_2_O, B: 0.1% CH_3_COOH in MeOH HRGC-Q-MS Mode: EI and SIM mode Column: capillary column	16 PAs/PA *N*-oxides	97–105	LOD: - LOQ: HPLC MS/MS: 1–50 µg/kg GC-MS 10 µg/kg	[76]
Honey	Solvent of extraction: LLE + 0.05 M of H_2_SO_4_ Clean-up process: SCX-SPE	HPLC-QTRAP-MS/MS Mode: +Ve ESI MRM Column: C18 at 25 °C M.P: A: 0.5% FA in H_2_O, B: 0.5% FA + 5% H_2_O in 94.5% MeOH	17 PAs/PA *N*-oxides	60–110	LOD: - LOQ: 1–3 µg/kg	[77]
Plant	Solvent of extraction: SLE by MeOH Clean-up process: -	LC-TOF-MS Column: C18 (150 mm × 2.1 mm i.d., 3 μm) M.P: 0.1% CH_3_COOH in H_2_O, B: 100% ACN	342 PAs/PA *N*-oxides	-	-	[78]
Plant	Solvent of extraction: closed system technique of microwave-assisted extraction Clean-up process: -	HPLC-diode array Wavelength: 220 nm LCQ-IT-MS Mode: +Ve ESI, SIM Column: C18(3.9 mm × 150 mm, 5 µm) M.P: A: 0.1% FA in 20 mM NH_4_CH_3_CO_2_, B: 0.1% FA in ACN	2 PAs/PA *N*-oxides	99–107	LOD MAE: 0.26 PHWE: 1.32 µg/g LOQ: MAE: 1.04 PHWE: 5.29 µg/g	[79]
Honey	Solvent of extraction: SCX-SPE followed by 2 reduction steps using zinc and LiAlH_4_ with subsequent sialylation Clean-up process: -	HRGC-MS Carrier gas: helium Column: DB-1MS fused-silica (30 m 60.32 mm) capillary column	2 (sum of retronecine and heliotridine)	80–86	LOD: - LOQ: 0.01 ppm	[80]
Plant	Solvent of extraction: 0.2% of HCL in an ultrasonic bath followed by centrifuge for 10 min then filtration through a 0.45 μm membrane Clean-up process: -	HPLC-IT-MS Mode: ESI Column: C18 (250 × 4.6 mm i.d; 5 μm) at 25°C M.P: A: 1% FA in H_2_O, B: 100% ACN	1 PAs	-	LOD: 0.5 ng/mL LOQ: 1 ng/mL	[81]
Plant	Solvent of extraction: 0.2% of HCL in an ultrasonic bath, using ammonium solution pH was adjusted to 9–10 and extracted using CHCl_3_ Clean-up process: -	HPLC-IT-MS Wavelength: 560 nm Mode: +Ve ESI Column: C18 (250 mm×4.6 mm, 5 µm) at 25 °C M.P: A: 1% FA in H_2_O, B: ACN	13 PAs/PA *N*-oxides	91–102	LOD: 0.26 nmol/mL LOQ: -	[82]

ACN: acetonitrile; AcOH: acetic acid; C18: octadecyl bonded silica; CHCl_3_: chloroform; DART: direct analysis in real time; DCM: dichloromethane; DLLME: dispersive liquid–liquid microextraction; DSHCSH: disodium hydrogen citrate sesquihydrate; dSPE: dispersive solid-phase extraction; ESI: electrospray ionization; EtOAc: ethyl acetate; FA: formic acid; GCB: graphitized carbon black; H_2_O: water; H_2_SO_4_: sulfuric acid; HCl: hydrochloric acid; HDMS^E^: high-definition MS^E^; HILIC: hydrophobic interaction liquid chromatography; HPLC: high performance liquid chromatography; HRMS: high resolution mass spectrometry; IM: ion mobility; iPrOH: isopropyl alcohol; IT: ion-trap LLE: liquid–liquid extraction; LOD: limit of detection; LOQ: limit of quantification; LP-MS-NH_2_: large pore mesostructured silica with amino groups; MAE: microwave-assisted extraction; MCX: mixed-mode cationic exchange; MeOH: methanol; MRM: multiple reaction monitoring; MS/MS: tandem mass spectrometry; MS: mass spectrometry; MSDP: matrix solid-phase dispersion; Na_2_CO_3_: sodium carbonate; Na_2_SO_4_: sodium sulphate; NaCL: sodium chloride; NH_3_:ammonia; NH_4_OH: ammonium hydroxide; PA *N*-oxides: pyrrolizidine alkaloids *N*-oxide; PAs: pyrrolizidine alkaloids; PFP: pentafluoro phenylpropyl column; PHWE: pressurized hot water extraction; PSA: primary secondary amine; PVDF: polyvinylidene difluoride; Q: single quadrupole; QTOF: quadrupole time-of-flight; QTRAP: hybrid triple quadrupole-linear ion trap; QuEChERs: quick, easy, cheap, effective, rugged, and safe; QuPPe: quick polar pesticides; RP-MS: chromatographic column based on core enhanced technology; SCX: strong cation exchange; SIM: selected ion monitoring; SLE: solid–liquid extraction; SPE: solid-phase extraction; TQ: triple quadrupole; TSCDH: trisodium citrate dihydrate; UHPLC: ultra-high-performance liquid chromatography.

**Table 4:** Selected PAs and PA-*N*-oxides parent ions (MS1) and daughter ions (MS2).

No.	Compound	MS1 ^a^	MS2 ^b^	DP ^c^	EP ^d^	CE ^e^	CXP ^f^	Reference
		(*m*/*z*)	(*m*/*z*)	(V)		(eV)	(V)	
1	Monocrotaline	326.2	121	53	10	28	45	[34]
		326.3	121.2	106	10	39	10	[105]
			121	131	10	41	10	[106]
			94.0	106	10	73	18	[105]
		326.1	120.1	161	10	43	8	[51]
			94.1	161	10	73	12	[51]
			194.1	161	10	39	12	[51]
2	Erucifoline	350.2	138	42	10	33	64	[34]
		350.2	94.0			40		[101]
		350.3	67.2	121	10	73	12	[106]
3	Monocrotaline NOs	342.2	137	38	10	34	53	[34]
			137.0	136	10	41	6	[105]
			120.1	136	10	51	6	[105]
			342.2	146	10	15	22	[106]
4	Europine	330.2	138	43	10	22	68	[34]
		330.4	138.1	66	10	31	10	[106]
5	Intermedine	300.1	94.1	96	10	33	12	[34]
			138.1	96	10	27	8	[51]
			156.0	96	10	37	10	[51]
		300.2	94.1	81	10	37	6	[51]
			138.1	81	10	31	6	[105]
		300.4	94.0	96	10	37	8	[105]
								[106]
6	Indicine	300.1	156	42	10	24	48	[34]
		300.5	94.1	91	10	37	8	[106]
7	Lycopsamine	300.2	156	52	10	39	48	[34]
		300.1	94.1	96	10	33	12	[51]
			138.1	96	10	27	8	[51]
			156.0	96	10	37	10	[51]
		300.2	138.2	60	10	30		[107]
			120.3	60	10	32		[107]
			138.1	91	10	29	8	[105]
			94.1	91	10	37	16	[105]
		300.5	94.0	86	10	37	8	[106]
8	Erucifoline NOs	366.2	118	16	10	33	48	[34]
		366.1	94.1	111	10	65	10	[106]
9	Europine NOs	346.2	256	25	10	25	75	[34]
			172.2	126	10	43	6	[106]
10	Intermedine NOs	316.3	172.2	56	10	37	14	[106]
11	Indicine NOs	316.2	172	28	10	31	68	[34]
		316.4	172.2	71	10	39	12	[106]
12	Lycopsamine NOs	316.2	172	42	10	37	47	[34]
		316.3	138.2	118	10	29		[107]
			94.0	118	10	44		[107]
		316.4	172.3	66	10	43	14	[106]
13	Retrorsine	352.3	120.1	116	10	43	8	[105]
			138.1	116	10	43	8	[105]
		352.2	120.0			30		[101]
		352.1	138.1	161	10	43	8	[51]
			119.2	161	10	73	12	[51]
			94.0	161	10	39	12	[51]
		352.2	138	45	10	47	41	[34]
		352.4	120.1	121	10	41	10	[106]
14	Trichodesmine	354.3	222.0	111	10	41	12	[105]
			120.1	111	10	53	6	[105]
		354.2	222	28	10	33	47	[34]
		354.3	222.1	121	10	39	14	[106]
15	Retrorsine NOs	368.3	94.1	111	10	73	16	[105]
			120.1	111	10	49	6	[105]
			94.0			40		[101]
		368.1	119.0	121	10	39	8	[51]
			94.1	121	10	71	6	[51]
			84.0	121	10	41	8	[51]
		368.2	118	38	10	37	64	[34]
		368.3	94.0	60	10	30	12	[106]
16	Seneciphylline	334.2	138	43	10	31	75	[34]
			120.0			39		[101]
			138.1	106	10	30	8	[105]
			120.1	106	10	39	10	[105]
		334.3	120.1	106	10	37	10	[106]
17	Heliotrine	314.2	156	35	10	26	48	[34]
			138.0			25		[101]
		314.3	138.1	76	10	31	8	[105]
			156.1	76	10	39	8	[105]
		314.2	138.2	86	10	29	10	[106]
18	Seneciphylline NOs	350.2	118	37	10	28	75	[34]
			120.0			30		[101]
			94.1	86	10	67	16	[105]
			118.1	86	10	45	6	[105]
		350.4	94.1	121	10	63	8	[106]
19	Heliotrine NOs	330.2	172	45	10	26	53	[34]
		330.3	172.2	71	10	39	12	[106]
20	Senecionine	336.2	120.0	121	10	41	20	[105]
			138.0	121	10	41	8	[105]
			120	27	10	33	42	[34]
			120			30		[101]
		336.1	120.1	136	10	37	8	[51]
			93.9	136	10	39	12	[51]
			91.1	136	10	77	14	[51]
		336.3	120.0	136	10	43	10	[106]
21	Senecivernine	336.2	120	43	10	28	46	[34]
		336.3	120.1	136	10	41	10	[106]
22	Senecionine NOs	352.3	94.2	91	10	67	6	[105]
			136.0	91	10	51	12	[105]
		352.2	120.0			30		[101]
			136	35	10	37	47	[34]
		352.1	120.1	156	10	39	6	[51]
			324.3	156	10	37	14	[51]
			93.9	156	10	41	12	[51]
		352.4	94.0	126	10	65	8	[106]
23	Senecivernine NOs	352.2	136	43	10	36	48	[34]
		352.4	94.0	131	10	63	8	[106]
24	Echimidine	398.2	220	23	10	24	54	[34]
			120.2	131	10	31	8	[51]
			220.1	131	10	23	10	[51]
			83.0	131	10	29	6	[51]
			120.3	75	10	35		[107]
		398.3	220.3	75	10	22		[107]
			120.0	76	10	35	8	[105]
			220.1	76	10	25	12	[105]
		398.2	120.0	111	10	33	10	[106]
25	Senkirkine	366.3	168.0	86	10	43	8	[105]
			150.0	86	10	39	8	[105]
		366.2	168	44	10	24	54	[34]
		366.1	168.2	96	10	39	12	[106]
26	Lasiocarpine	412.2	220	53	10	22	67	[34]
		412.3	120.1	96	10	39	10	[106]
27	Lasiocarpine NOs	428.2	254	75	10	30	38	[34]
		428.4	94.1	111	10	69	6	[106]
28	Jacobine	352.2	155	47	10	34	47	[34]
29	Jacobine NOs	368.2	296	36	10	26	45	[34]
30	Spartioidine	334.2	120.0			30		[101]
31	Integerrimine	336.2	120.0			30		[101]
32	Integerrimine NOs	352.2	120.0			30		[101]
33	Jacozine	350.2	94.0			40		[101]
34	Riddelliine	350.2	94.0			40		[101]
35	Riddelliine NOs	366.2	94.0			40		[101]
36	Jacobine	352.2	120.0			30		[101]
37	Jacobine NOs	368.2	94.0			40		[101]
38	Jacoline	370.2	120.0			30		[101]
39	Jacoline NOs	386	94.0			40		[101]
40	Acetylseneciphylline	376.2	120.0			30		[101]
41	Acetylseneciphylline NOs	392.2	120.0			30		[101]
42	Jaconine	388.2	120.0			30		[101]
43	Jaconine NOs	404.2	94.0			40		[101]
44	Acetylerucifoline	392.2	120.0			40		[101]
45	Acetylerucifoline NOs	408.2	94.0			40		[101]
46	Acetyllycopsamine	342.3	198.4	53	10	38		[107]
			138.3	53	10	36		[107]
			120.2	53	10	36		[107]
			94.2	53	10	60		[107]
47	Echimidine NOs	414.2	352	42	10	21	75	[34]
		414.4	396.4	80	10	35		[107]
			254.0	80	10	41		[107]
48	Echiumine	382.5	220.3	51	10	25		[107]
			120.3	51	10	38		[107]
49	Echiumine NOs	398.3	220.4	80	10	22		[107]
			120.2	80	10	35		[107]
50	7,9-Ditigloylretronecine NOs	336.0	138.2	60	10	42		[107]
			120.2	60	10	42		[107]

a—precursor ion, b—product ion, c—declustering potential, d—entrance potential, e—collision energy, f—collision cell exit potential, NOs (PA *N*-oxides).

## Data Availability

Not applicable.
